# Interactions between microenvironment and cancer cells in two animal models of bone metastasis

**DOI:** 10.1038/sj.bjc.6604238

**Published:** 2008-02-05

**Authors:** S Blouin, M F Baslé, D Chappard

**Affiliations:** 1 Inserm, U922–LHEA, Faculté de Médecine, Angers Cedex 49045, France

**Keywords:** bone metastasis, osteolysis, osteoclastogenesis, osteoclast, microenvironment

## Abstract

The preferential proliferation of cancer cells in the bone microenvironment is poorly characterised. Expression pattern of bone marrow and other organ microenvironment in contact with osteolytic (Walker W256) and osteoblastic (MatLyLu MLL) metastases were investigated. Fisher and Copenhagen rats received, respectively, W256 and MLL cells injection. Bone and soft tissues were analysed by immunochemistry for DKK1, cathepsin K, RANKL, MCSF or IL6 expression. Tartrate-resistant acid phosphatase (TRAcP)-positive cells were detected by a histoenzymatic technique. In bone, expressions of MCSF and DKK1 were shown in stromal cells of the bone marrow, in contact with metastatic foci of both tumours. Many stromal cells were found RANKL positive in the vicinity of the tumours. Cells expressing cathepsin K and multinucleated TRAcP+ cells were found in direct contact with trabeculae but also in bone marrow spaces near metastatic cells. In extraosseous tumours, cells in contact with malignant cells did not expressed DKK1, MCSF, cathepsin K and IL6. Some RANKL+ cells were found in the periphery of subcutaneous tumours but may represent Langerhans cells. Abnormal presence of TRAcP+ cells was never observed in the vicinity of malignant cells. Interaction between stromal and cancer cells induces the expression on the formers of characteristics leading to osteoclastogenesis only in the bone microenvironment.

Bone is a preferential site for metastasis in different types of cancer. Bone metastases induce an increased morbidity (pain, fractures, nerve compression, hypercalcaemia…) and compromise the long-term survival. They occur in approximately 65–75% of patients with an advanced breast or prostate cancer and 15–40% of patients with a carcinoma of the lung, bladder, skin or kidney. The median survival after first recurrence of breast cancer in bone is 20 months (Coleman, 2001). Once a tumour has metastasised to bone, it is virtually incurable and only palliative therapies can be proposed (Mundy, 1997).

Interactions between the bone marrow environment and cancer cells were first advocated by Paget, who described them as ‘the seed and the soil’ theory (cited by Guise and Mundy, 1998): bone provides the ‘fertile soil’ in which certain cancer cell (‘seeds’) find favourable conditions for growth. Mineralised bone matrix is known to represent a rich storehouse of growth factors that are mobilised by osteoclastic resorption and become active in the local microenvironment. The release of these growth factors constitutes one of the key mechanisms in the bone remodelling allowing the physiological coupling between osteoblasts (the bone forming cells) and the osteoclasts (bone-resorbing cells) (Mundy *et al*, 2001). When localised in the marrow spaces, tumour cells, in turn, secrete additional factors that interfere with bone remodelling leading to tissular changes that characterise the osteolytic or osteoblastic expression of the metastases. Thus, local interactions between tumour and bone cells form a vicious cycle that underlies the development of skeletal metastases. During the last decade, it has become evident that interactions between tumour and bone cells may change the phenotype of both populations.

Breast and prostate cancer are the two tumour types that most commonly metastasise to bone. On one hand, bone has characteristics that allow the selective homing of tumour cells: (1) a release of attractive molecules for these particular types of cancer (stromal cell-derived factor-1, epidermal growth factor, collagen degradation products, low glycosylated osteonectin…) inducing specific chemotaxis (Cooper *et al*, 2003); (2) the expression of adhesion molecule on the endothelial surface of bone marrow capillaries (due to a variety of integrins such as *α*4*β*1, *α*5*β*1, *α*v*β*3, *α*v*β*5) (Roodman, 2003); (3) appropriate growth factors and extracellular matrix proteins present in the bone marrow microenvironment (parathyroid hormone-related protein (PTHrP), tumour growth factor beta (TGF-*β*), vascular endothelium growth factor (VEGF)…) (Woodhouse *et al*, 1997; Guise and Mundy, 1998; Keller *et al*, 2001). On the other hand, these peculiar tumour cells express proteins that favour propensity to migrate and anchor in bone: this includes chemotaxis (CXCR4) (Muller *et al*, 2001), extravasation and bone marrow homing (integrins, E-cadherin) (Woodhouse *et al*, 1997), pericellular proteolysis and invasion (MMPs and ADAMs) (Woodhouse *et al*, 1997; Zigrino *et al*, 2005), angiogenesis (Woodhouse *et al*, 1997), osteoclastogenesis (Roodman, 2004), growth factor regulation (follistatin) (Risbridger *et al*, 2001; Reis *et al*, 2004) and extracellular matrix alteration (proteoglycan-1) (Timar *et al*, 2002). A multigenic study of breast cancer metastasis to bone has provided evidences for a causal role of IL-11, connective tissue-derived growth factor (CTGF) and CXCR4, along with osteopontin in osteolytic metastases. The combined overexpression of these genes gives the transfected cells a metastatic activity close to that of the highly aggressive cell populations endogenously expressing the entire bone metastasis gene set (Kang *et al*, 2003).

Bone metastases are commonly characterised as osteolytic or osteoblastic from a radiological point of view. This represents two extremes of a continuum in which dysregulation of the normal bone remodelling process occurs and alters bone mass and bone microarchitecture. Breast cancer is most often associated with osteolytic metastases, whereas osteoblastic metastases are common in prostate cancer. Osteolytic lesions are due to a marked increase in osteoclast number with a reduced osteoblastic activity. Parathyroid hormone-related protein is a major mediator between tumour and bone cells and induces the osteolytic process through the RANKL pathway (Guise, 2000). On the contrary, osteoblastic metastases are characterised by a dramatic increase in osteoformation, but always possess a resorption component (Mundy, 2002; Logothetis and Lin, 2005).

Most researches have focused on the interactions between tumour cells and osteoclasts or osteoblasts. However, interactions with surroundings cells have been poorly investigated. Because osteoblast precursors are stromal cells (present in the marrow spaces) that interact with haematopoietic osteoclast precursors via the RANK/RANKL/osteoprotegerin system (Thomas *et al*, 1999), the potential influence of stromal cells on metastatic cells needs to be investigated.

The aim of this study was to induce metastases in the rat to investigate the expression pattern of these key molecules in the bone microenvironment and also in the stroma of other organs invaded by a metastasis. Two cell lines known to induce either osteolytic or osteoblastic metastases were used.

## MATERIALS AND METHODS

### Cell lines and culture conditions

Two malignant cell lines capable of inducing bone metastases were used in the present study.

#### Walker 256/B cells

The Walker 256/B (W256) cells (issued of a rat mammary carcinoma) are associated with osteolytic metastases (Wingen *et al*, 1986; Rizzoli and Fleisch, 1987; Blouin *et al*, 2005). W256 cells (kindly provided by Pr R Rizzoli, Geneva, Switzerland) were grown in Dulbecco's modified Eagle's medium (DMEM; Eurobio, Les Ulis, France) containing 10% heat-inactivated fetal calf serum (FCS; Seromed Biochrom, Berlin, Germany), 100 UI ml^−1^ of penicillin (Eurobio), 100 *μ*g ml^−1^ of streptomycin sulphate (Eurobio), 1% of non essential amino acids (NEAA; Cambrex, Walkersville, MD, USA) and 1 mM of sodium pyruvate (Eurobio). Cells were cultured at 37°C in a humidified incubator with 5% CO_2_. To obtain cells with a bone trophicity, 10^7^ cells were serially passaged intraperitoneally at 7-day intervals in Fisher rats to obtain malignant ascite.

#### MatLyLu cells

The MatLyLu cell line is an androgen-independent, highly metastatic and anaplastic tumour of rat origin, which spreads to the lymph nodes and invariably to the lung. Metastases are reported to be osteoblastic in the vertebrae (Vieweg *et al*, 1994). The MatLyLu tumour cell line was obtained from ECCAC (European Collection of Animal Cell Cultures, Salisbury, Wiltshire, UK). MatLyLu cells were grown in Roswell Park Memorial Institute 1640 medium (RPMI 1640; Eurobio) containing 10% heat-inactivated fetal calf serum (FCS; Seromed Biochrom), 100 UI ml^−1^ of penicillin (Eurobio), 100 *μ*g ml^−1^ of streptomycin sulphate (Eurobio) and 250 nM of dexamethasone (Sigma, Saint Quentin Fallavier, France). Cells were cultured at 37°C in a humidified incubator with 5% CO_2_.

### Animals

The Animal Care and Use committee at the University of Angers approved all procedures. Nine female Fisher F344/NHsd rats (Harlan, Gannat, France), 3 weeks old were maintained for 6 months in the local vivarium conditions (24°C and a 12 h light/dark cycle). Nine female Copenhagen born from COP/NCrl rats (Charles River, Sulzfeld, Germany), bred in our University animal facilities were maintained for 6 months in the same conditions. Rats were given a standard laboratory food (UAR, Villemoison sur Orge, France) and water *ad libitum*.

F344 rats were anaesthetised with isoflurane (AErrane; Baxter S.A, Belgium) and received an intracardiac injection of 10^7^ W256 cells in 0.25 ml saline. They also received intramuscular (quadriceps femoris) and subcutaneous (base of the neck) injection with 10^7^ W256 cells.

Copenhagen rats were anaesthetised with isoflurane and received an intracardiac injection of 3.25 × 10^4^ MLL cells in 0.25 ml saline. They also received intramuscular, intraliver, intraspleen and subcutaneous injections with 3.25 × 10^4^ MLL cells in the same locations than above. For both types of rats, the cell number to be injected was determined as the most appropriate in preliminary experiments. Data were compared with results obtained on control rats.

Rats were sacrificed by asphyxiation with CO_2_, 9 days (F344) or 15 days (COP) after the intracardiac injection of tumour cells. These durations have been determined by preliminary studies and were found sufficient to obtain bone metastases before animals die from other tumour localisation. The hindlimbs were carefully dissected; bones and other tissue samples (liver, spleen, brain, subcutaneous and intramuscular nodules) were placed in a formalin-alcohol-based fixative at +4°C during 24 h.

### Histological staining methods on paraffin sections

Paraffin sections (4 *μ*m) were deparaffinised and stained by Haematoxylin Phloxine Saffron (HPS) as a standard tissue stain. Immunohistochemistry was performed on femur and organs of six F344 and six COP rats. Bone decalcification was done using a ready-to-use decalcifying fluid according to the manufacturer's recommendations (Surgipath, Labonord, Templemars, France). Bone and other tissues were then dehydrated, embedded in paraffin and sectioned at 4 *μ*m. Antigen retrieval was performed by bain-marie heating in a citrate buffer (pH 6). Immunochemistry was performed using DAKOCYTOMATION procedure with the Dako autostainer automat (Dako, Glostrup, Denmark). The following proteins were traced using specific antibodies:

- Dickkopf-1 (Dkk-1) is an antagonist of the canonical Wnt signalling by binding directly to the LDL receptor-related protein 5/6 (LRP5/6). The interaction between DKK1 ant LRP5/6 induces an inhibition of bone formation (Johnson *et al*, 2004). The reagent was a purified goat polyclonal antibody (Tebu-bio, sc-14949) used at a dilution of 1 : 50. Positivity is characterised by a cell membrane labelling.
- Interleukin 6 (IL-6) is a pleitropic cytokine. Its production by bone marrow stromal cells enhances osteoclastogenesis (with subsequent bone resorption) and tumour cell growth (Roodman, 2003). We used a purified goat polyclonal antibody (Tebu-bio, sc-1265) at a dilution of 1 : 50. Positivity is characterised by a cytoplasmic labelling.
- RANKL is produced by osteoblasts and stromal cells and induces osteoclastogenesis by interacting with its receptor at the osteoclastic surface (Roodman, 2003). We used a purified goat polyclonal antibody (Tebu-bio, sc-7628) at a dilution of 1 : 200. Positivity is characterised by a cell membrane labelling.
- Cathepsin K is a cysteine protease highly expressed by osteoclasts and is implicated in the bone matrix collagen breakdown (Yasuda *et al*, 2005). The purified goat polyclonal antibody (Tebu-bio, sc-6507) was used at a dilution of 1 : 200. Positivity is characterised by a cytoplasmic labelling.
- Macrophage-colony stimulating factor (M-CSF) is expressed by stromal cells and osteoblasts. It is required together with RANKL to induce the development of mature osteoclasts (Boyle *et al*, 2003). The purified goat polyclonal antibody (Tebu-bio, sc-1324) was used at a dilution of 1 : 200. Positivity is characterised by a cytoplasmic labelling.

Control-positive tissues were obtained for each antibody. Sections were counterstained with haematoxylin.

### TRAcP cells detection

Tartrate-resistant acid phosphatase (TRAcP)-positive cells were detected by a histoenzymatic technique in three F344 and three COP rats. Tartrate-resistant acid phosphatase is a key enzyme found in high level in osteoclasts; it is involved in the intracellular degradation of collagen and noncollagenous proteins of the bone matrix (Vaaraniemi *et al*, 2004). Tissues were embedded in methylmethacrylate at 4°C to maintain enzyme activity. Bone samples were embedded undecalcified. Sections (7-*μ*m-thick) were cut dry on a heavy-duty microtome equipped with 50° tungsten carbide knives (Leica Polycut S, Rueil-Malmaison, France). Tartrate-resistant acid phosphatase was revealed by a simultaneous coupling reaction using 1-naphtyl phosphate (Sigma-Aldrich Chemical, Illkirsh, France) as substrate and Fast Violet B as the diazonium salt. Sections were counterstained with phosphomolybdic aniline blue (Chappard *et al*, 1983). Tartrate-resistant acid phosphatase-positive cells appeared with a brownish tint. Collagen fibres densely packed in the form of bone matrix or in the tissue stroma appear with a blue tint.

## RESULTS

### Tumour growth in animals

F344 rats developed tumours at subcutaneous and intramuscular site of injection. They also developed pulmonary, liver, brain and bone tumours secondary to the intracardiac injection of W256 cells. In the femur, osteolytic foci were preferentially localised below the growth cartilage and formed a large and irregular band filled with malignant cells.

Copenhagen rats similarly developed tumours at subcutaneous, intramuscular, liver and spleen sites of injection. They also developed pulmonary and bone tumours after the intracardiac injection. In the femur, focal areas of osteolysis were disseminated all over the metaphysis. No osteosclerotic lesions were found.

### Histological and immunohistochemical characteristics of bone tumours

**Figure 1:**
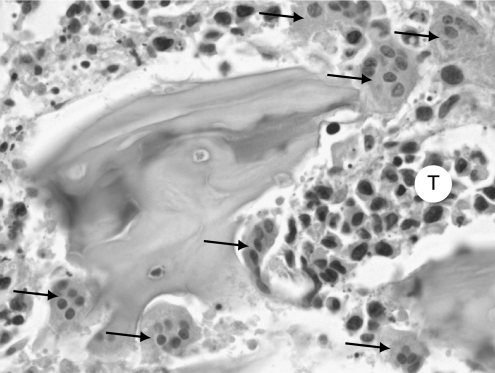
Increased osteoclast number (arrows) and resorption lacunae on trabeculae near metastatic foci of tumour cell (T) in bone (HPS staining). Original magnification: × 400.

Tumour cells appeared most often as closely packed nodules ill-demarcated from the haematopoeitic marrow. Some tumoral cells were also found isolated in bone marrow. For both line cells, tumour cells were large with a high nuclear/cytoplasmic ratio, they exhibited proeminent nucleoli and a basophilic cytoplasm without vacuoles. Numerous mitoses were observed. An increased osteoclastogenesis was assessed in the immediate vicinity of the tumour foci, leading to a dramatic increase of active erosion surfaces and the resorption of complete bone trabeculae (Figure 1). Siderophages (macrophages containing brownish cytoplasmic particles) were frequently found, they reflect the necrotic and remodelling steps occurring in the haematopoietic bone marrow secondary to tumour invasion (Weinberg, 1983). These cells were positive with the Perls reaction for iron. They are readily distinguishable from immunolabelled cells under the microscope, although they can appear with a similar intensity on black and white microphotographs.

**Figure 2:**
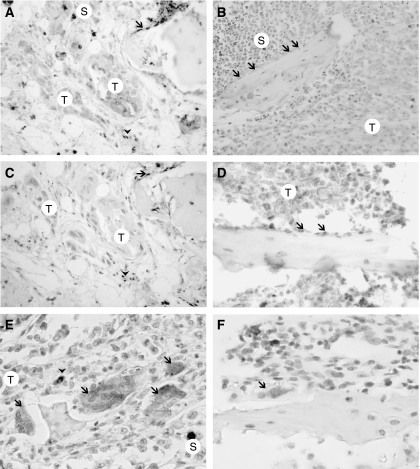
(**A**) Dkk-1 expression in cells distant from tumoral foci (T) in bone with osteoblasts (arrow) and stromal cells (arrow head) exhibiting membrane and cytoplasmic labelling. Other cells appearing in black at the top of the image are siderophages (S). (**B**) the negative control section stained with the same concentration of control antibody, note that a group of osteoblasts (arrows) is negative. (**C**) RANKL expression near tumour foci in bone. Osteoblasts (arrow) and stromal cells (arrow head) are RANKL+. Tumour foci are RANKL negative. (**D**) The negative control section stained with the same concentration of control antibody, note that a group of osteoblasts (arrow) is negative. (**E**) Cathepsin K expression near tumoral foci (T) in bone. Cathepsin K+ multi- and mononucleated cells (arrow head) have a diffuse and faint cytoplasmic labelling. Both can be found in marrow spaces at a distance from bone surfaces. A siderophage (iron-loaded macrophage) is also evidenced (S). (**F**) The negative control section stained with the same concentration of control antibody; note that an osteoclasts (arrow) is negative. For all images, the original magnification is × 400.

Both models exhibited the same immunohistochemical labelling pattern: tumour cells never exhibited significant labelling for M-CSF, Dkk-1, RANKL, IL-6 or cathepsin K (Figure 2). A strong membrane expression of M-CSF was found in most bone marrow stromal cells in contact with the metastatic foci. Dkk-1 was also expressed at high levels in stromal cells and osteoblasts in contact with metastatic foci in both models (Figure 2A). At distance, there was no significant labelling for Dkk-1 in these cell populations. Many stromal cells showed RANKL positivity in the vicinity of the tumour (Figure 2C). RANKL labelling was also seen on osteoblasts at distance of the tumour foci. These stromal cells (which were labelled by Dkk1, M-CSF and RANKL) were found at distance from trabeculae: they were mononucleated with often a large granular cytoplasm; some of them appeared spindle-shaped. The IL-6 detection was not conclusive due to a nonspecific label induced by the prior decalcification.

Two types of cells were found to express a faint and homogenous cytoplasmic positivity for cathepsin K: typical multinucleated osteoclasts at the surface of bone trabeculae and multi- or mononucleated cells (either round or with cytoplasmic branches) in the marrow spaces. These cells were mixed with haematopoietic cells and were at a distance from bone surfaces (Figure 2E); they concentrate in the close vicinity of the tumour foci and at distance, on the contrary, they appeared scantily distributed.

**Figure 3:**
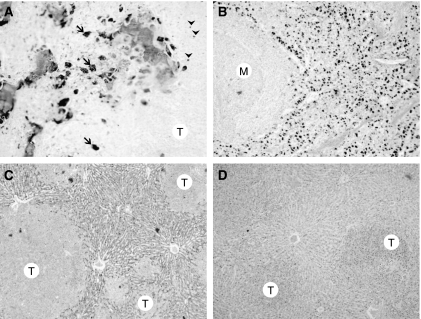
(**A**) Multinucleated TRAcP+ cells in bone are apposed on trabeculae. In bone marrow spaces, multi- (arrow) and mononucleated (arrow head) TRAcP+ cells are also encountered far from trabecular surfaces and near tumour cells (T) (undecalcified plastic section). Original magnification: × 200. (**B**) TRAcP+ cells are found interspersed in the spleen parenchyma in the presence or not of tumour cells. These mononucleated TRAcP+ cells are round with a large cytoplasm in the red pulp. Note that the white pulp is negative (M=Malpighi corpuscle). (plastic section). Original magnification: × 50. (**C**) IL-6 expression in the liver. Note that tumoural foci (T) are negative, whereas hepatocytes show a homogeneous granular cytoplasmic expression. Original magnification: × 50. (**D**) The negative control section stained with the same concentration of control antibody, note that an osteoclasts (arrow) is negative. Original magnification: × 50.

Two types of cells exhibited TRAcP positivity: multinucleated cells apposed on trabeculae and either multi- or mononucleated cells encountered in bone marrow spaces were; they were situated far from trabecular surfaces and in the close vicinity of metastatic cells, their density decreased at distance from tumoral foci (Figure 3A).

### Histological and immunohistochemical characteristics of extraosseous tumours

Tumour nodules of both types of carcinomas were dense and well delimited by a fibrous capsule. There were composed of a large majority of tumour cells mixed with less numerous stromal cells. Tumour cells showed a different aspect according to the metastatic site: brain metastases were composed of spindle-shaped cells, whereas in other sites, they appeared round or polygonal (especially with Walker cells). For both line cells, tumour cells were large with a high nucleocytoplasmic ratio; prominent nucleoli were often present and the cytoplasms were devoid of vacuoles. Numerous mitoses were observed, but small foci of necrosis were also noted inside nodules.

Both tumour models exhibited the same immunohistochemical labelling pattern: tumour cells never exhibited any significant labelling for M-CSF, Dkk-1, RANKL, IL-6 and cathepsin K. Stromal cells in contact with malignant cells did not express Dkk-1, M-CSF and cathepsin K. At a distance from tumour foci, they also failed to express these markers. IL-6 expression was found homogeneous in the liver parenchyma with diffused granular cytoplasmic expression in the hepatocytes. The staining was found in all the liver parenchyma and not restricted to the areas surrounding the tumour. Few stromal cells were located around the tumoral foci and did not exhibit IL-6 expression (Figure 3C). Some RANKL-positive stromal cells were found at the periphery of subcutaneous tumours. These mononucleated cells were round or stellate with an abundant cytoplasm. No RANKL+ cells were found in any other extraosseous site.

Tartrate-resistant acid phosphatase-positive cells were found interspersed in the spleen parenchyma whether tumour cells were present or not. These mononucleated TRAcP+ cells appeared round with a large granular cytoplasm; the nucleus was ovoid or reniform (Figure 3B). Tartrate-resistant acid phosphatase-positive cells were never observed in the vicinity of malignant cells in any other extraosseous metastases.

## DISCUSSION

It is an oversimplification to consider only osteoblasts and osteoclasts as the interacting partners of tumours cells in the vicious cycle of cancer bone metastases. In addition to a variety of matrix components, the tumour stroma contains a rich cell population, which includes fibroblasts, smooth muscle cells, endothelial cells, dendritic cells, macrophages and other inflammatory cells (Zigrino *et al*, 2005). This forms a network with a complex crosstalk influencing cellular activities. For example, various actors of the tumour microenvironment, such as fibroblasts, immune cells and the extracellular matrix, influence the ability of TGF-*β* to promote or suppress carcinoma progression and metastasis (Bierie and Moses, 2006). In a previous work, we have shown an influence of the microenvironment on tumour cell in the 5T2 multiple myeloma murine model, as a high bone remodelling (induced by ovariectomy) dramatically increased tumour growth (Libouban *et al*, 2003). This altered microenvironment was able to select a highly aggressive myeloma cell line (Libouban *et al*, 2004). Others have also reported that parathyroid hormone injection in athymic mice induced a preferential localisation of PC-3 prostate cancer cells in bones (Schneider *et al*, 2005).

Interactions of malignant cells with endothelial cells in bone metastases have been previously investigated. van der Pluijm *et al* (2001) observed significantly elevated mRNA levels for VEGF-A and -B in bone metastases compared with soft tissue metastases induced by the human breast cancer cells MDA-MB-231. Di Raimondo *et al* (2000) similarly detected higher concentrations of the angiogenic peptides (VEGF, bFGF (basic fibroblast growth factor) and HGF (hepatocyte growth factor)) in bone marrow than in the peripheral circulation of patients with multiple myeloma. This suggests the specific induction within the bone microenvironment of angiogenic factors, which allows the development of a neovascular network favouring the tumour growth.

Interaction between tumour cells and other stromal components also plays a major role in cancer progression. Cancer cells can alter the surrounding connective tissues and modulate the metabolism of fibroblasts, thus resulting in the production of a collagenous stroma that supports the tumoral process. Fibroblast is a key component of tumours, because it acquires a modified phenotype similar to those associated with wound healing. Such ‘activated’ fibroblasts have been coined ‘carcinoma-associated fibroblasts’ (Kalluri and Zeisberg, 2006). Carcinoma-associated fibroblasts are implicated in extracellular matrix production, which provides a lattice that facilitates cancer progression; they are one of the main sources of VEGF, thus providing more nutriment to the metastastic cells (Kalluri and Zeisberg, 2006). Shimamura *et al* (2005) have investigated the microenvironment of osteolytic metastases caused by MDA-MB 231 breast carcinoma cells in nude mice. At 2 weeks after an intracardiac injection with MDA-MB 231 cells, foci of tumour cells were associated with osteoclastogenesis together with angiogenesis related to intense the expression of VEGF (Shimamura *et al*, 2005). In our study, we have also found specific modifications in the bone microenvironment around the tumour foci with expression of M-CSF, Dkk-1 and RANKL in non-osteoblastic cells. Moreover, mononucleated TRAcP+ cells were also encountered in bone marrow spaces far from trabecular surfaces. This denotes an increased induction of osteoclast precursors, as mononuclear TRAcP+ cells in the bone marrow are a source for mature multinucleated osteoclasts (Baron *et al*, 1986). These mononucleated TRAcP+ cells expressed cathepsin K and were found in medullar spaces at distance from bone surfaces. All these cells were found with a density decreasing, as the distance from tumour cells increased. It is probable that a gradient of cytokines released from malignant cells can explain this finding. Tumour cells produce a variety of soluble factors (PTHrP, TGF-*β*, IGF, endothelin-1…) that are employed as a paracrine communication system with surrounding cells or as an autocrine regulation system (Guise and Mundy, 1998; Guise *et al*, 2003; Lee *et al*, 2003). W256 and MatLyLu cells are unable to resorb bone by themselves and have been reported to produce high levels of PTHrP, which promotes osteoclastogenesis (Rizzoli and Fleisch, 1987; Rabbani *et al*, 1999).

The stromal expression of important cytokines involved in the bone remodelling was found altered only when metastatic cells were developing in a bony microenvironment. No expression of these cytokines could be observed in the tumour stroma when metastastic cells had developed in other organs. The few RANKL+ cells found at the periphery of subcutaneous tumours were probably Langerhans cells. Tartrate-resistant acid phosphatase-positive cells encountered both in invaded and non-invaded spleens probably corresponded to activated macrophages, a condition previously noted by others (Hayman *et al*, 2000). Tartrate-resistant acid phosphatase-positive cells were previously observed in the stroma of bone tumours (Yam *et al*, 1987). Tartrate-resistant acid phosphatase-positive osteoclast-like cells are also observed in giant cells tumours that can develop within bone (and cause osteolytic lesions) or soft tissues (Teot *et al*, 1996). In this tumour, the malignant component is of fibroblastic origin and express RANKL, which provokes osteoclastogenesis (Murata *et al*, 2005). In our models, stromal cells were able to express RANKL and to induce osteoclastogenesis only in the bone compartment.

Tumour cells do not have the same cytokine pattern of expression according to their location and can modulate it depending on the surrounding stromal cells. For example, breast cancer cells have an increased expression of PTHrP in bone metastases when compared with other sites (Mundy, 2002). As a consequence, the microenvironment can also influence the tumour cells: Walker cells acquired a spindle shape only when located in brain metastases, whereas they appeared round or polygonal in other sites. The difference may also be supported by soft tissue stromal cells, which can have different surface receptors than bone stromal cells, making them unable to respond to tumour cell stimulation.

In conclusion, interaction between stromal and cancer cells alters the expression of the former leading to osteoclastogenesis in the bone microenvironment. These modifications allow the growth of the metastases in a convenient microenvironment. It thus explains the preferential localisation observed in our bone metastasis models. The major role of osteoclasts and osteoblasts in bone metastasis formation has led to underestimate the critical function of microenvironment in metastasis growth.

## References

[bib1] Baron R, Neff L, Tran Van P, Nefussi JR, Vignery A (1986) Kinetic and cytochemical identification of osteoclast precursors and their differentiation into multinucleated osteoclasts. Am J Pathol 122: 363–378 3946557 PMC1888102

[bib2] Bierie B, Moses HL (2006) Tumour microenvironment: TGFbeta: the molecular Jekyll and Hyde of cancer. Nat Rev Cancer 6: 506–520 16794634 10.1038/nrc1926

[bib3] Blouin S, Basle MF, Chappard D (2005) Rat models of bone metastases. Clin Exp Metastasis 22: 605–614 16670964 10.1007/s10585-006-9002-5

[bib4] Boyle WJ, Simonet WS, Lacey DL (2003) Osteoclast differentiation and activation. Nature 423: 337–342 12748652 10.1038/nature01658

[bib5] Chappard D, Alexandre C, Riffat G (1983) Histochemical identification of osteoclasts. Review of current methods and reappraisal of a simple procedure for routine diagnosis on undecalcified human iliac bone biopsies. Basic Appl Histochem 27: 75–85 6193776

[bib6] Coleman RE (2001) Metastatic bone disease: clinical features, pathophysiology and treatment strategies. Cancer Treat Rev 27: 165–176 11417967 10.1053/ctrv.2000.0210

[bib7] Cooper CR, Chay CH, Gendernalik JD, Lee HL, Bhatia J, Taichman RS, McCauley LK, Keller ET, Pienta KJ (2003) Stromal factors involved in prostate carcinoma metastasis to bone. Cancer 97: 739–747 12548571 10.1002/cncr.11181

[bib8] Di Raimondo F, Azzaro MP, Palumbo G, Bagnato S, Giustolisi G, Floridia P, Sortino G, Giustolisi R (2000) Angiogenic factors in multiple myeloma: higher levels in bone marrow than in peripheral blood. Haematologica 85: 800–805 10942925

[bib9] Guise TA (2000) Molecular mechanisms of osteolytic bone metastases. Cancer 88: 2892–2898 10898330 10.1002/1097-0142(20000615)88:12+<2892::aid-cncr2>3.0.co;2-y

[bib10] Guise TA, Mundy GR (1998) Cancer and bone. Endocr Rev 19: 18–54 9494779 10.1210/edrv.19.1.0323

[bib11] Guise TA, Yin JJ, Mohammad KS (2003) Role of endothelin-1 in osteoblastic bone metastases. Cancer 97: 779–784 12548575 10.1002/cncr.11129

[bib12] Hayman AR, Bune AJ, Bradley JR, Rashbass J, Cox TM (2000) Osteoclastic tartrate-resistant acid phosphatase (Acp 5): its localization to dendritic cells and diverse murine tissues. J Histochem Cytochem 48: 219–228 10639488 10.1177/002215540004800207

[bib13] Johnson ML, Harnish K, Nusse R, Van Hul W (2004) LRP5 and Wnt signaling: a union made for bone. J Bone Miner Res 19: 1749–1757 15476573 10.1359/JBMR.040816

[bib14] Kalluri R, Zeisberg M (2006) Fibroblasts in cancer. Nat Rev Cancer 6: 392–401 16572188 10.1038/nrc1877

[bib15] Kang Y, Siegel PM, Shu W, Drobnjak M, Kakonen SM, Cordon-Cardo C, Guise TA, Massague J (2003) A multigenic program mediating breast cancer metastasis to bone. Cancer Cell 3: 537–549 12842083 10.1016/s1535-6108(03)00132-6

[bib16] Keller ET, Zhang J, Cooper CR, Smith PC, McCauley LK, Pienta KJ, Taichman RS (2001) Prostate carcinoma skeletal metastases: cross-talk between tumor and bone. Cancer Met Rev 20: 333–349 10.1023/a:1015599831232 12085970

[bib17] Lee Y, Schwarz E, Davies M, Jo M, Gates J, Wu J, Zhang X, Lieberman JR (2003) Differences in the cytokine profiles associated with prostate cancer cell induced osteoblastic and osteolytic lesions in bone. J Orthop Res 21: 62–72 12507581 10.1016/S0736-0266(02)00095-5

[bib18] Libouban H, Moreau MF, Basle MF, Bataille R, Chappard D (2003) Increased bone remodeling due to ovariectomy dramatically increases tumoral growth in the 5T2 multiple myeloma mouse model. Bone 33: 283–292 13678768 10.1016/s8756-3282(03)00196-0

[bib19] Libouban H, Moreau MF, Basle MF, Bataille R, Chappard D (2004) Selection of a highly aggressive myeloma cell line by an altered bone microenvironment in the C57BL/KaLwRij mouse. Biochem Biophys Res Commun 316: 859–866 15033480 10.1016/j.bbrc.2004.02.131

[bib20] Logothetis CJ, Lin SH (2005) Osteoblasts in prostate cancer metastasis to bone. Nat Rev Cancer 5: 21–28 15630412 10.1038/nrc1528

[bib21] Muller A, Homey B, Soto H, Ge N, Catron D, Buchanan ME, McClanahan T, Murphy E, Yuan W, Wagner SN, Barrera JL, Mohar A, Verastegui E, Zlotnik A (2001) Involvement of chemokine receptors in breast cancer metastasis. Nature 410: 50–56 11242036 10.1038/35065016

[bib22] Mundy GR (1997) Mechanisms of bone metastasis. Cancer 80: 1546–1556 9362421 10.1002/(sici)1097-0142(19971015)80:8+<1546::aid-cncr4>3.3.co;2-r

[bib23] Mundy GR (2002) Metastasis to bone: causes, consequences and therapeutic opportunities. Nat Rev Cancer 2: 584–593 12154351 10.1038/nrc867

[bib24] Mundy GR, Chen D, Zhao M, Dallas S, Xu C, Harris S (2001) Growth regulatory factors and bone. Rev Endocr Metab Disord 2: 105–115 11704973 10.1023/a:1010015309973

[bib25] Murata A, Fujita T, Kawahara N, Tsuchiya H, Tomita K (2005) Osteoblast lineage properties in giant cell tumors of bone. J Orthop Sci 10: 581–588 16307183 10.1007/s00776-005-0946-0

[bib26] Rabbani SA, Gladu J, Harakidas P, Jamison B, Goltzman D (1999) Over-production of parathyroid hormone-related peptide results in increased osteolytic skeletal metastasis by prostate cancer cells *in vivo*. Int J Cancer 80: 257–264 9935208 10.1002/(sici)1097-0215(19990118)80:2<257::aid-ijc15>3.0.co;2-3

[bib27] Reis FM, Luisi S, Carneiro MM, Cobellis L, Federico M, Camargos AF, Petraglia F (2004) Activin, inhibin and the human breast. Mol Cell Endocr 225: 77–82 10.1016/j.mce.2004.02.016 15451571

[bib28] Risbridger GP, Mellor SL, McPherson SJ, Schmitt JF (2001) The contribution of inhibins and activins to malignant prostate disease. Mol Cell Endocr 180: 149–153 10.1016/s0303-7207(01)00497-x 11451585

[bib29] Rizzoli R, Fleisch H (1987) The Walker 256/B carcinosarcoma in thyroparathyroidectomized rats: a model to evaluate inhibitors of bone resorption. Calcif Tissue Int 41: 202–207 2960427 10.1007/BF02555239

[bib30] Roodman GD (2003) Role of stromal-derived cytokines and growth factors in bone metastasis. Cancer 97: 733–738 12548570 10.1002/cncr.11148

[bib31] Roodman GD (2004) Mechanisms of bone metastasis. N Eng J Med 350: 1655–1664 10.1056/NEJMra030831 15084698

[bib32] Schneider A, Kalikin LM, Mattos AC, Keller ET, Allen MJ, Pienta KJ, McCauley LK (2005) Bone turnover mediates preferential localization of prostate cancer in the skeleton. Endocrinology 146: 1727–1736 15637291 10.1210/en.2004-1211

[bib33] Shimamura T, Amizuka N, Li M, Freitas PH, White JH, Henderson JE, Shingaki S, Nakajima T, Ozawa H (2005) Histological observations on the microenvironment of osteolytic bone metastasis by breast carcinoma cell line. BiomedRes 26: 159–172 10.2220/biomedres.26.159 16152732

[bib34] Teot LA, O'Keefe RJ, Rosier RN, O'Connell JX, Fox EJ, Hicks DG (1996) Extraosseous primary and recurrent giant cell tumors: transforming growth factor-beta1 and -beta2 expression may explain metaplastic bone formation. Hum Pathol 27: 625–632 8698303 10.1016/s0046-8177(96)90389-5

[bib35] Thomas RJ, Guise TA, Yin JJ, Elliott J, Horwood NJ, Martin TJ, Gillespie MT (1999) Breast cancer cells interact with osteoblasts to support osteoclast formation. Endocrinology 140: 4451–4458 10499498 10.1210/endo.140.10.7037

[bib36] Timar J, Lapis K, Dudas J, Sebestyen A, Kopper L, Kovalszky I (2002) Proteoglycans and tumor progression: Janus-faced molecules with contradictory functions in cancer. Semin Cancer Biol 12: 173–186 12083848 10.1016/S1044-579X(02)00021-4

[bib37] Vaaraniemi J, Halleen JM, Kaarlonen K, Ylipahkala H, Alatalo SL, Andersson G, Kaija H, Vihko P, Vaananen HK (2004) Intracellular machinery for matrix degradation in bone-resorbing osteoclasts. J Bone Miner Res 19: 1432–1440 15312243 10.1359/JBMR.040603

[bib38] van der Pluijm G, Sijmons B, Vloedgraven H, Deckers M, Papapoulos S, Lowik C (2001) Monitoring metastatic behavior of human tumor cells in mice with species-specific polymerase chain reaction: elevated expression of angiogenesis and bone resorption stimulators by breast cancer in bone metastases. J Bone Min Res 16: 1077–1091 10.1359/jbmr.2001.16.6.1077 11393785

[bib39] Vieweg J, Rosenthal FM, Bannerji R, Heston WD, Fair WR, Gansbacher B, Gilboa E (1994) Immunotherapy of prostate cancer in the Dunning rat model: use of cytokine gene modified tumor vaccines. Cancer Res 54: 1760–1765 8137291

[bib40] Weinberg ED (1983) Iron in neoplastic disease. Nutr Cancer 4: 223–233 6302639 10.1080/01635588209513761

[bib41] Wingen F, Eichmann T, Manegold C, Krempien B (1986) Effects of new bisphosphonic acids on tumor-induced bone destruction in the rat. J Cancer Res Clin Oncol 111: 35–41 3949849 10.1007/BF00402773 PMC12253181

[bib42] Woodhouse EC, Chuaqui RF, Liotta LA (1997) General mechanisms of metastasis. Cancer 80: 1529–1537 9362419 10.1002/(sici)1097-0142(19971015)80:8+<1529::aid-cncr2>3.3.co;2-#

[bib43] Yam LT, Janckila AJ, Li CY, Lam WK (1987) Cytochemistry of tartrate-resistant acid phosphatase: 15 years' experience. Leukemia 1: 285–288 3669748

[bib44] Yasuda Y, Kaleta J, Bromme D (2005) The role of cathepsins in osteoporosis and arthritis: rationale for the design of new therapeutics. Adv Drug Deliv Rev 57: 973–993 15876399 10.1016/j.addr.2004.12.013

[bib45] Zigrino P, Loffek S, Mauch C (2005) Tumor-stroma interactions: their role in the control of tumor cell invasion. Biochimie 87: 321–328 15781319 10.1016/j.biochi.2004.10.025

